# Repeatability and comparison of 2D and 4D flow MRI measurement of intracranial blood flow and pulsatility in healthy individuals and patients with cerebral small vessel disease

**DOI:** 10.3389/fpsyg.2023.1125038

**Published:** 2023-05-30

**Authors:** Alasdair G. Morgan, Michael J. Thrippleton, Michael Stringer, Ning Jin, Joanna M. Wardlaw, Ian Marshall

**Affiliations:** ^1^Centre for Clinical Brain Sciences, University of Edinburgh, Edinburgh, United Kingdom; ^2^UK Dementia Research Institute, University of Edinburgh, Edinburgh, United Kingdom; ^3^Edinburgh Imaging, University of Edinburgh, Edinburgh, United Kingdom; ^4^Siemens Medical Solutions USA, Inc., Cleveland, OH, United States

**Keywords:** flow imaging, pulsatility, intracranial vessels, 4D flow, phase-contrast, MRI

## Abstract

**Introduction:**

While 2D phase-contrast MRI is often used to examine intracranial vessels in neurovascular disease contexts, the ability of 4D flow to assess many vessels at once makes it an attractive alternative. We aimed to assess the repeatability, reliability, and conformity of 2D and 4D flow across intracranial vessels.

**Methods:**

Using correlation analyses and paired *t*-tests, test-retest repeatability, intra-rater reliability, and inter-method conformity for measurements of pulsatility index (PI) and mean flow were assessed in the arteries and veins of 11 healthy volunteers. Inter-method conformity was also assessed in 10 patients with small vessel disease.

**Results:**

Repeatability for PI measurements was mostly classed as good using both 2D (median ICC = 0.765) and 4D (0.772) methods, and for mean flow was mostly moderate across both (2D: 0.711, 4D: 0.571). 4D reliability was good for PI (0.877–0.906) and moderate for mean flow (0.459–0.723). Arterial PI measurements were generally higher using the 2D method, while mean flow was mostly higher using 4D flow.

**Discussion:**

These results imply that PI measurement using 4D flow is repeatable and reliable across intracranial arteries and veins, but care should be paid to absolute flow measurements as they are susceptible to variation depending on slice placement, resolution, and lumen segmentation practices.

## Introduction

Arterial stiffening is believed to be an underlying mechanism in several neurological pathologies, including stroke (Sutton-Tyrrell et al., 2005), Alzheimer’s disease (AD) (Sabayan et al., 2012) and cerebral small vessel disease (SVD) (Poels et al., 2012). Due to factors such as loss of elastin in the vessel walls, ageing, diabetes, hypertension, arterial remodelling (wall thickening) and smooth muscle dysfunction, blood vessels lose compliance and thus are thought to be less able to regulate blood flow (Lee and Oh, 2010). With stiffened vessel walls, systemic pulsation is less dampened and therefore thought to expose the brain to strong pulsations. Furthermore, higher intracranial venous pulsatility has been associated with more advanced AD (Rivera-Rivera et al., 2017) and SVD (Shi et al., 2018a)—which may represent compensation to maintain steady intracranial pressure (Rivera-Rivera et al., 2017) or possibly transmitted arterial pulsation (Shi et al., 2018a). This suggests that intracranial veins should be considered alongside arteries when conducting cerebrovascular research on these pathologies.

Vessel stiffness and flow pulsatility have been measured using various techniques in clinical and research contexts. Pulse wave velocity (PWV) between two vascular locations is used as a measure of vessel stiffness, as stiffer walls absorb less energy and are thus thought to allow faster transmission of the waveform (Boutouyrie et al., 2009). Transcranial Doppler (TCD) ultrasound is a relatively inexpensive way of measuring blood flow velocities primarily in the middle cerebral artery (MCA), predominantly through the temporal bone insonation window. Meanwhile, 2D phase-contrast magnetic resonance imaging (2D PC-MRI) can provide non-invasive measurement of both flow velocity in and cross-sectional area of any major cerebral vessel. This means pulsatility can be calculated using volumetric blood flow values over the cardiac cycle, not just velocities. This technique has proven to be robust and reliable (Zananiri et al., 1993; Enzmann et al., 1994; McCauley et al., 1995; Hoppe et al., 1998) but requires a separate scan for each 2D plane along any vessels of interest. In recent years, a “4D flow” MRI technique has been developed that encodes velocity in three orthogonal directions, in three spatial dimensions and over time. This technique assesses a volume of tissue, removing the requirement to place individual 2D imaging planes perpendicularly to each vessel of interest, and allows for *post hoc* analysis of vessels captured within the imaging volume. This ability to assess flow in any direction at multiple locations throughout the acquired volume means that spatial differences in cerebral blood flow and pulsatility transmission or attenuation can more easily be examined than with 2D PC-MRI. Furthermore, clinical applications of neurovascular 4D flow MRI have been demonstrated in recent years in areas such as MCA bypass surgery (Sekine et al., 2021) and arteriovenous malformations (AVMs) (Takeda et al., 2021).

Due to the large amounts of data collected, 4D flow MRI scans often require acceleration with various techniques to counteract long scan times (and therefore reduce the risk of movement artefact) which can both lower the signal-to-noise ratio (SNR) compared to the 2D method (Markl et al., 2012) as well as lead to temporal blurring (Terada et al., 2022). Investigation of intracranial vessels is limited by spatial resolution, and reducing the voxel size can increase scan time and reduce SNR further. 4D flow and 2D PC-MRI have been compared previously in intracranial arteries (Holmgren et al., 2019) (*N* = 35 elderly subjects, age 70–91 years) and the repeatability of both has been measured intracranially [2D: arteries in 22 healthy volunteers and 8 post-stroke patients, age 23–69 years (Chang et al., 2020), 4D: arteries and one vein in 10 healthy volunteers, age 22–33 years (Wen et al., 2019)]. Here we have addressed not only the conformity and repeatability of a wider selection of intracranial vessels (including veins which are often overlooked) using multiple acquisition methods and tests, but also similarities and differences in methods and flow parameters between healthy volunteers and patients with stroke presentations of SVD.

In this work, we performed a prospective study to assess the test-retest repeatability, intra-rater reliability, and inter-method conformity of 2D PC and 4D flow MRI in measuring flow- and stiffness-related parameters in several major intracranial arteries and veins. We also aimed to qualitatively assess the feasibility of these techniques in a population of clinically-relevant patients, as well as compare these flow and stiffness parameters between patient and healthy volunteer groups.

## Materials and methods

### Subjects

We recruited 11 healthy volunteers (mean age 31.6 ± 11.5 years, 63.6% males) without health issues from the Edinburgh area, regardless of age or sex, of whom 10 had complete MRI data across both visits. One subject did not complete their second scan session (specifically, the 4D flow scan) due to a technical issue.

For the patient subgroup, we recruited 11 patients (mean age 60.0 ± 11.1 years) from the Mild Stroke Study 3 (MSS3), a prospective study of stroke presentations of sporadic SVD that recruited patients with lacunar ischaemic stroke or mild cortical stroke from NHS Lothian clinical stroke services. One of these patients exhibited a lot of movement and was unable to tolerate the scanner following the previous sequences (unrelated to this work) and withdrew before the flow scans took place. We subsequently scanned a new subject so as to reach the desired N of 10.

The MSS3 rationale and design has been previously published (Clancy et al., 2021). The volunteer and MSS3 studies were approved by the South East Scotland Research Ethics Committee (REC 14/HV/001 and 18/SS/0044, respectively). Written informed consent was obtained from all participants.

### Imaging protocol

We scanned each healthy volunteer across two sessions within a single visit to assess repeatability, and each SVD patient in one session to assess 2D-4D differences.

**Figure 1:**
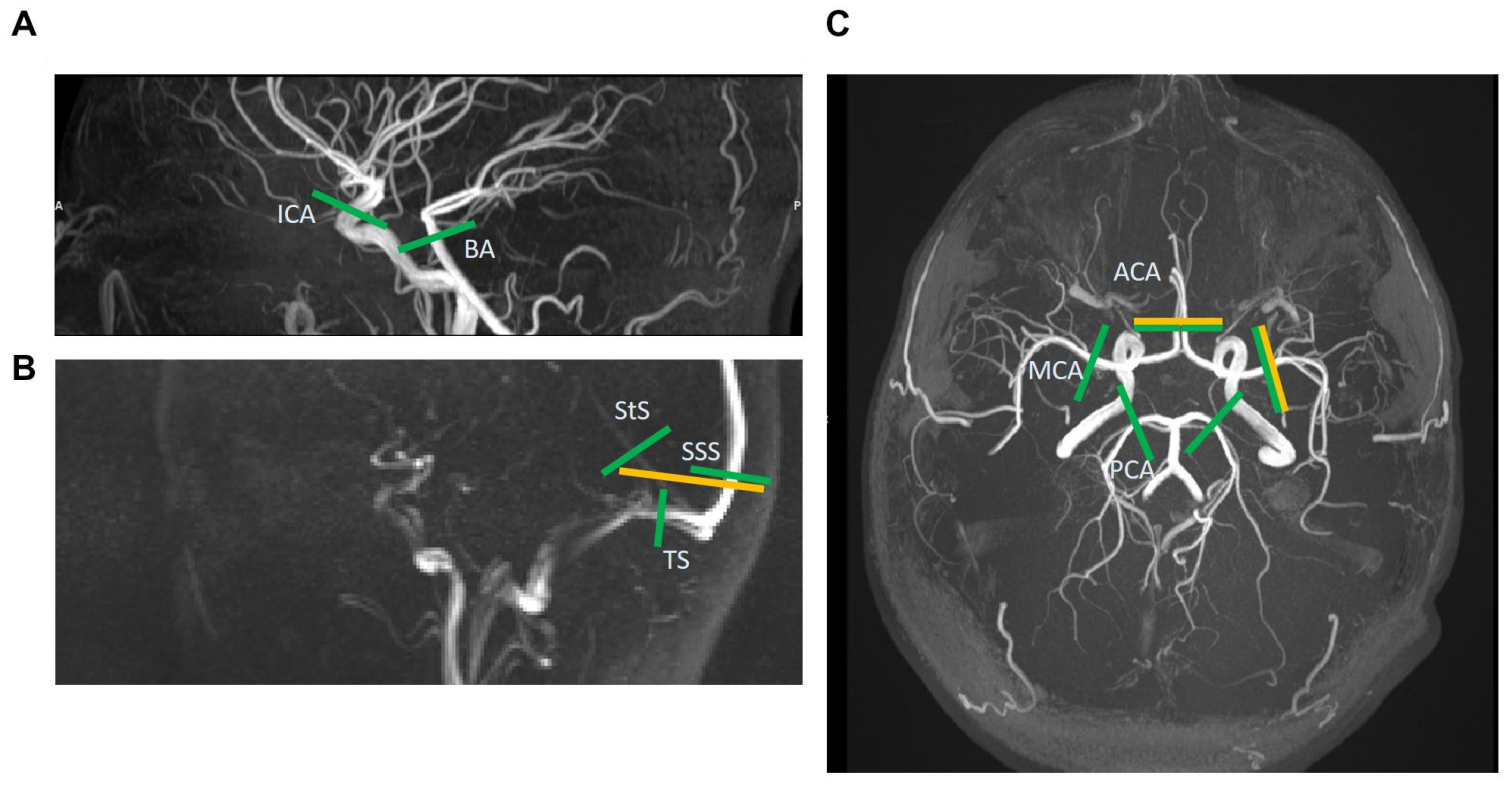
Slice locations of 2D phase-contrast MR images (yellow) and those extracted post-hoc from the 4D flow volume (green). A total of five vessels are assessed with 2D and 13 with 4D. **(A)** This sagittal time-of-flight (TOF) image shows the internal carotid and basilar artery slices (ICA & BA). **(B)** This sagittal low-res vessel scout image shows the venous sinuses more clearly than the TOF. Here the 2D slice across the superior sagittal and straight sinuses (SSS & StS) can be seen. These two vessels, as well as the slices across the transverse sinuses (TS), are shown for the 4D image too. **(C)** This axial TOF image shows the 2D slices for the right middle cerebral artery (MCA) and combined anterior cerebral artery (ACA) images, as well as the 4D slices for the ACAs, MCAs, and posterior cerebral arteries (PCA).

During each visit, we imaged participants on the same 3 T MRI scanner (MAGNETOM Prisma, Siemens Healthcare, Erlangen, Germany; *syngo* MR Numaris 4 VE11C) using a 32-channel phased-array head coil. We also acquired a time-of-flight (TOF) sequence to collect a high-resolution volume covering the major cerebral arteries. We acquired three 2D PC-MRI scans the “gold-standard,” well-established (Shi et al., 2018a; Blair et al., 2020, 2021; Clancy et al., 2021) method of *in-vivo*, non-invasive blood flow quantification (Figure 1) perpendicular to the (i) first segment of the right MCA, (RMCA) (TR/TE: 10.34/6.17 ms, temporal resolution: 20.68 ms, flip angle (FA): 12°, *v*_enc_: 80 cm/s), (ii) the second segment of the anterior cerebral arteries (ACAs) (TR/TE = 10.44/6.24 ms, temporal resolution: 20.88 ms, FA: 12°, *v*_enc_: 70 cm/s) and (iii) venous sinuses [superior sagittal sinus (SSS) and straight sinus (StS)] (TR/TE: 10.94/6.62 ms, temporal resolution: 21.88 ms, FA: 12°, *v*_enc_: 50 cm/s) with retrospective pulse triggering using a pulse oximeter and reconstruction into 32 timeframes. The venous slice was placed across both veins mentioned, as perpendicular to both as possible when anatomy allowed, otherwise the larger vessel (SSS) took precedent. Table 1 shows the sequence parameters.

**Table 1:** 2D PC-MRI and 4D flow sequence parameters.

Sequence parameter	2D phase-contrast MRI			4D flow
	RMCA	ACAs	Veins	
Acquired temp. Resolution (ms)*	20.68	20.88	21.88	87.68
TR (ms)	10.34	10.44	10.94	5.48
TE (ms)	6.17	6.24	6.62	2.85
Flip angle (°)	12			7
*v*_enc_ (cm/s)	80	70	50	100
Reconstructed cardiac time frames	32			25
Number of slices	1			40 (foot-head)
Field of view (mm) (phase-encoding × frequency-encoding)	235 × 176 (ant-post × foot-head)	191 × 240 (foot-head × left-right)	171 × 240 (left-right × ant-post)	180 × 224 (left-right × ant-post)
Phase encoding steps**	278	230	240	180
Bandwidth (Hz/pixel)	205	200	205	485
Spatial resolution (mm)	0.846 × 0.846	0.833 × 0.833	0.714 × 0.714	1 × 1
Slice thickness (mm)	3.10			1
Signal averages	1			1
Views per segment	1			4
Acquisition time (mins)	01:48–02:46	01:31–02:37	01:38–02:33	11:10–17:54
Acceleration factor	1			3 (PE direction, left-right)

*Temporal resolution defined as the time between successive acquisitions of the same line of k-space. **Not including acceleration factor.

During each session, following the 2D scans, subjects also underwent a 4D flow scan using a prototype pulse sequence (WIP 785B) covering the Circle of Willis and major cerebral arteries and veins (volume 224 × 180 × 40 mm^3^ with oblique-axial orientation, TR/TE: 5.48/2.85 ms, temporal resolution: 87.68 ms, FA: 7°, *v*_enc_: 100 cm/s) with retrospective pulse triggering. Data were reconstructed into 25 timeframes per cardiac cycle. The relatively small FA of 7° was chosen due to the short TR of the sequence and thus the need to prevent saturation and increase signal. We used GRAPPA (GeneRalised Autocalibrating Partial Parallel Acquisition) to speed up data acquisition, with undersampling occurring in the in-plane phase-encoding direction (acceleration factor 3). See Table 1 for more information. Both 2D and 4D scans were performed on each subject per session, with two sessions occurring in the same day (and a break of 5–10 min between) for the healthy volunteer subgroup. We recorded heart rate (bpm) during each session so as to assess any differences that may affect measurement of flow and pulsatility (Wahlin et al., 2013).

These 4D parameters were determined based on sequence optimisation on a previous group of healthy volunteers, wherein scan duration, image quality, and 4D-2D conformity were assessed. These parameters provided the optimal balance of these factors.

### Image processing

#### 2D phase-contrast MRI

Following a previously described approach (Shi et al., 2018a), one image analyst with two years’ experience manually drew regions of interest (ROIs) around the RMCA, ACAs, SSS and StS on maximum (over the cardiac cycle) magnitude or mean velocity phase-contrast images for each subject, using FSLeyes (version 6.0.1. Oxford, UK: FMRIB Centre; 2021). We carried out correction for background phase error by placing “background” ROIs close to the vessel and subsequently calculating the mean velocity within each background ROI to subtract it from the pixel velocities of the appropriate vessel. Using in-house Matlab (version 9 (R2018b), Natick, MA: The MathWorks Inc.; 2018) code (available at https://github.com/agm21ed), mean flow (ml/s) across the cardiac cycle was calculated for each vessel. Any phase wraparound caused by velocities above *v*_enc_ or below −*v*_enc_ was corrected by adjusting any measured velocities equal to or above the median velocity + 1*v_enc_ by − 2*v_enc,_ and any velocities equal to or below the median − 1*v_enc_ by +2*v_enc_, accordingly at each timeframe.

#### 4D flow MRI processing

**Figure 2:**
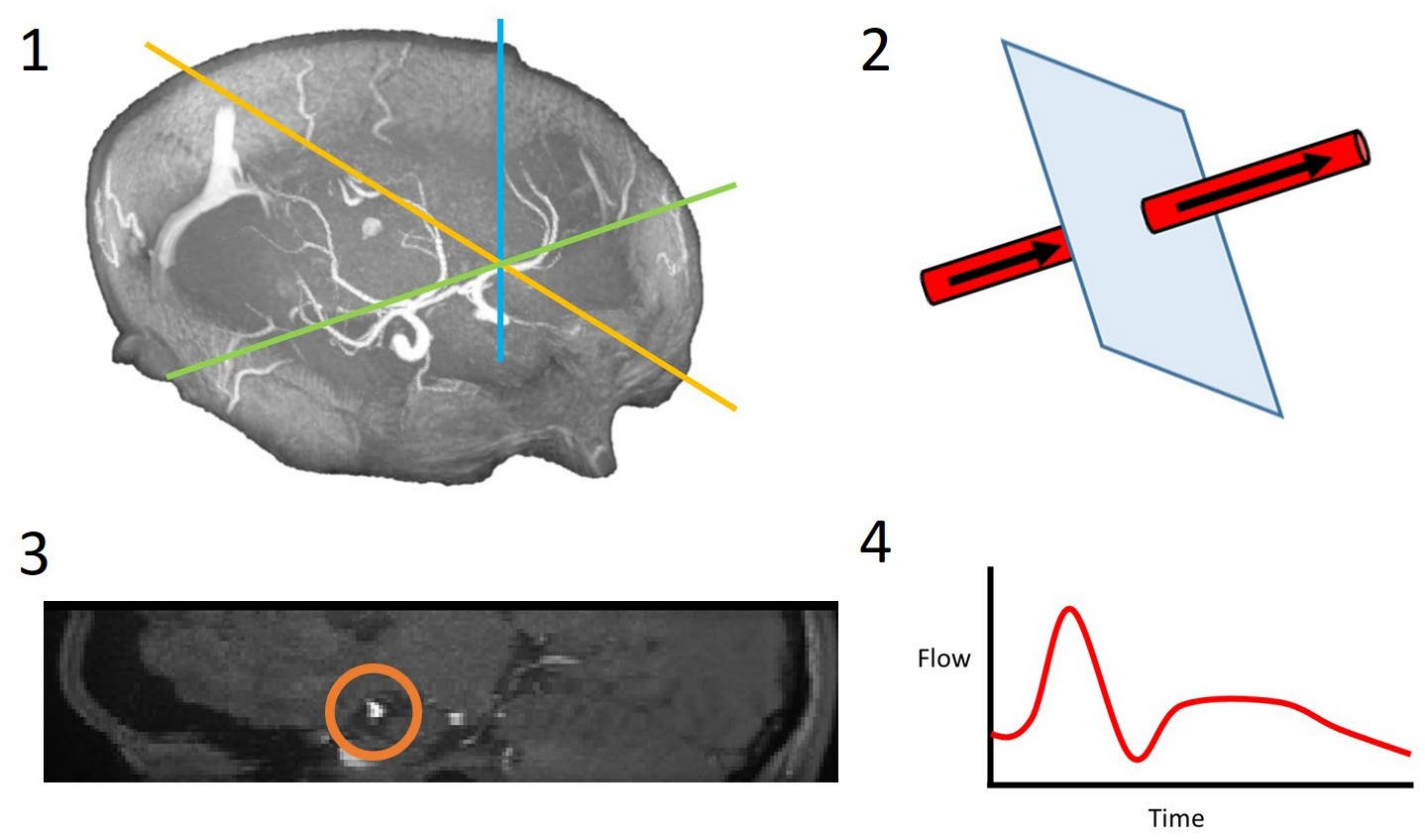
4D flow processing pipeline. 3D coordinates of the desired slice along the vessel of interest were saved (1); these coordinates and a vector along the vessel were used to slice the vessel perpendicularly (2); the resulting 2D slice allowed for the vessel lumen to be segmented (3); the velocities within this region of interest were extracted over all timeframes and a flow waveform was calculated (4).

We viewed the volumetric magnitude image produced by each 4D flow MRI (hereafter referred to as 4D flow) scan using ImageJ’s (Bethesda, Maryland, United States^1^) Volume Viewer plug-in. We examined the 3D vasculature of each subject, extracting the desired centre point coordinates within each vessel of interest and a vector along a section of each vessel from that point (Figure 2). We then used in-house Matlab code and a slicing function (*Extract Slice from Volume*^2^, MATLAB Central File Exchange, retrieved June 11, 2021, author username: pangyuteng) to extract slices perpendicular to all vessels of interest within the 4D volume. We drew ROIs around the vessel lumens and background tissue on these slices and used Matlab code to extract flow values (as for the 2D processing method). Thirteen vessels were assessed using this method (Figure 1): both right and left MCAs, anterior, and posterior cerebral arteries (ACAs and PCAs respectively), internal carotid arteries (ICAs), the basilar artery (BA), the SSS, StS, and both right and left transverse sinuses (TSs).

We used dot product vector multiplication to obtain the velocity component contributing to flow along the vessel [Equation (1)].

(1) $$v_{\mathrm{parallel}}=v\cdot u$$

where v is the velocity vector (*v_x_, v_y_, v_z_*) of a given pixel and u is the unit vector along the vessel. As with 2D processing, the velocities of all pixels within the vessel ROI were summed and multiplied by the pixel area to calculate flow (ml/s) for each timeframe.

We kept flow plane/slice locations consistent between methods and visits: ICA (C2-C4 segment), BA (~2 cm below PCA branch), MCA (M1 segment), ACA (A2 – postcommunicating part), PCA (P2 – postcommunicating part), SSS (occipital part), StS, TS (all venous locations ~2 cm from confluence of sinuses). In some cases, positioning of the 4D flow volume meant these desired locations along the vessels were not all acquired and therefore the closest vessel segments were used in these cases.

### Flow parameters

We calculated mean flow (ml/s) and an adapted Gosling’s (Gosling and King, 1974) pulsatility index (PI = (flow_max_ − flow_min_)/flow_mean_) for each vessel examined. By using the lumen-wide volumetric flow rates to calculate PI, rather than calculating PIs in individual pixels and taking the average of these, we reduced the potential effects of noise and obtained an arguably better representation of overall blood flow pulsatility in each given vessel. With the 4D flow data, we compared the sum of mean flow in the combined ICAs and BAs (“inflow”) with the sum of mean flow in the combined ACAs, MCAs, and PCAs (“cerebral flow”) so as to assess conservation of mass and therefore the precision of the sequence.

### Intra-rater reliability

To determine intra-rater reliability in the healthy volunteers, we processed a subset of data consisting of an artery (RMCA) and a vein (SSS) a second time (approximately 6 months after the first rating) for each subject using the 2D and 4D scans, as described above.

### Statistics

We assessed test-retest repeatability and intra-rater reliability in the healthy volunteer group using paired sample *t*-tests, intraclass correlation coefficients (ICC) and Bland-Altman analysis. Koo and Li’s grading system (Koo and Li, 2016) was used to interpret ICC scores: <0.5: poor, 0.5–0.75: moderate, 0.75–0.9: good, >0.9: excellent. For both healthy volunteer and patient groups, we assessed conformity between 2D and 4D results using paired sample *t*-tests, Bland-Altman analysis, and Pearson *r* correlation. *P*-values < 0.05 were considered statistically significant. We subsequently carried out a secondary analysis wherein both groups were combined for greater statistical power. Mean flow and PI measurement differences between healthy and patient groups were assessed using paired sample *t*-tests.

To assess the effect of vessel size on the test-retest repeatability error, we used Pearson’s *r* to calculate correlations between lumen cross-sectional area and the absolute mean difference in flow parameters between scan sessions.

## Results

### Subject characteristics

Of the subjects who completed the scanning session, nearly all lay still with minimal movement during the flow scans (mean 4D flow scan duration: 15:03 ± 1:47 min for healthy volunteer group and 14:18 ± 1:46 min for patient group) and no discernible movement artefacts or blurring were seen on the associated images. However, one patient displayed noticeable movement during the scan.

### Test-retest repeatability

For both 4D and 2D scans, flow waveforms were generally visually similar in shape between visits, but the absolute flow values were more susceptible to variation. Recorded heart rates showed and a mean (±SD) difference between visits of 3.6 ± 5.0 bpm, reflecting a percentage difference of 5.3 ± 7.5% relative to the visit 1 measurements (inter-visit correlation *r* = 0.79). We consider these differences acceptably small.

**Figure 3:**
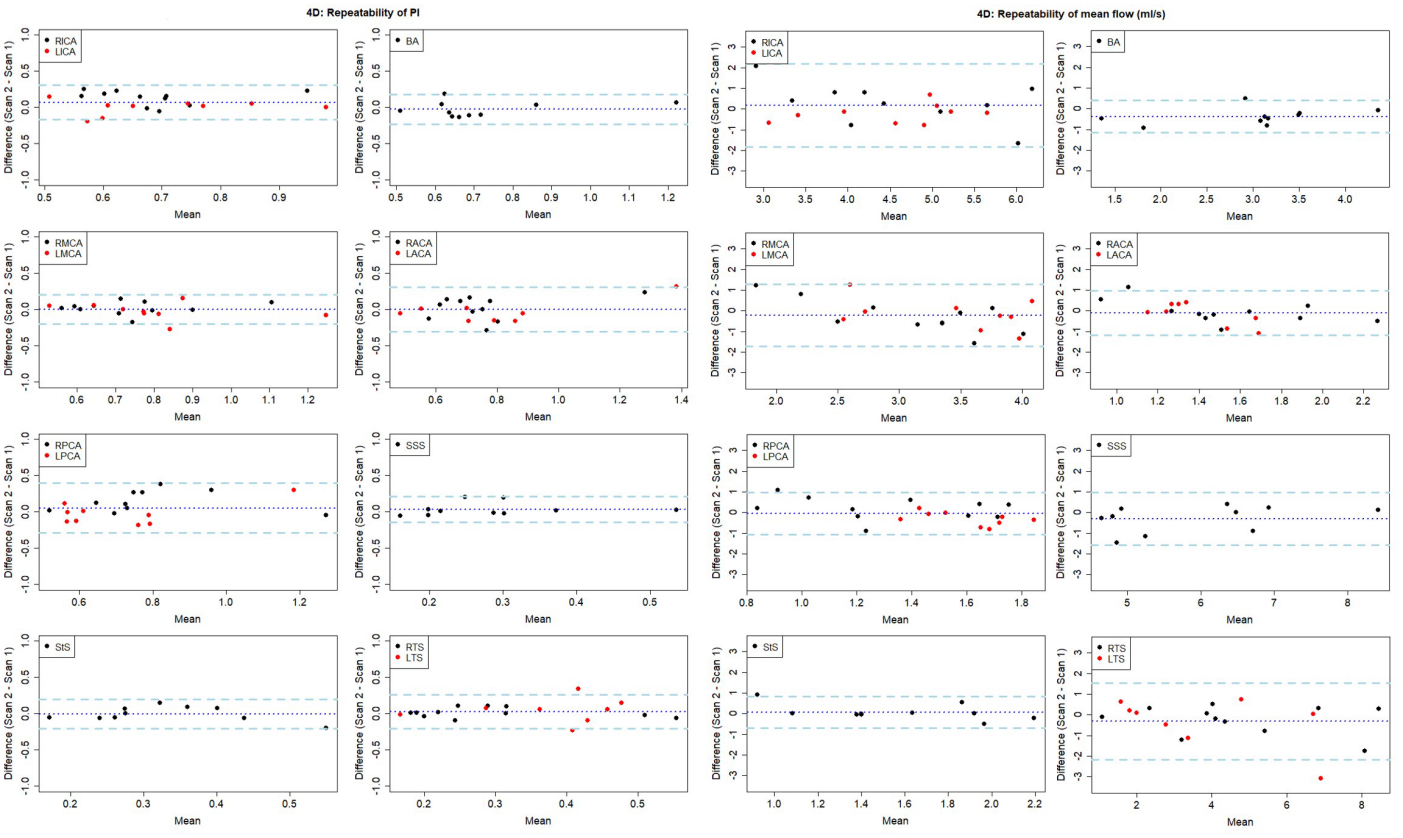
Bland-Altman plots showing visit-to-visit repeatability of PI and mean flow measurements using 4D flow. Central dotted line indicates mean difference between visits, outer dashed lines indicate 95% limits of agreement. RICA/LICA, right/left internal carotid artery; BA, basilar artery; RMCA/LMCA, right/left middle cerebral artery; RACA/LACA, right/left anterior cerebral artery; RPCA/LPCA, right/left posterior cerebral artery; SSS, superior sagittal sinus; StS, straight sinus; RTS/LTS, right/left transverse sinus.

The test-retest repeatability results for PI and mean flow measurements across both 2D and 4D scans are presented in Tables 2, 3, respectively. The 2D scans showed good PI repeatability (median ICC: 0.765, range 0.345–0.937; Table 2), except for the ACAs (0.345 and 0.571 for right and left ACAs, respectively), and moderate-to-good mean flow repeatability overall (median ICC: 0.711, range 0.674–0.964; Table 3). The 4D scans showed good PI repeatability overall (median ICC: 0.772, range: 0.226–0.912; Table 2), with poor ICCs seen in certain vessels (RICA: 0.432, RPCA: 0.526, and LTS: 0.226), and moderate mean flow repeatability (median ICC: 0.571, range: −0.160 to 0.946; Table 3) (Figure 3).

**Table 2:** Test-retest repeatability of PI.

Vessel	Intra-class correlation coefficient		Mean difference (s2-s1)		Standard deviation of difference	
	2D	4D	2D	4D	2D	4D
RICA	–	0.432 (poor)	–	0.12* (20.5%)	–	0.10 (17.1%)
LICA	–	0.703** (moderate)	–	0.02 (6.3%)	–	0.13 (24.9%)
BA	–	0.872*** (good)	–	−0.02 (−2.8%)	–	0.11 (16.8%)
RMCA	0.780*** (good)	0.912*** (excellent)	0.07 (7.4%)	0.04 (5.8%)	0.16 (16.5%)	0.06 (8.9%)
LMCA	–	0.808*** (good)	–	−0.04 (−3.5%)	–	0.01 (14.2%)
RACA	0.345 (poor)	0.794*** (good)	0.07 (14.1%)	0.05 (7.47%)	0.29 (38.0%)	0.13 (17.3%)
LACA	0.571* (moderate)	0.801** (good)	−0.04 (−1.3%)	−0.06 (−7.9%)	0.21 (25.7%)	0.17 (16.8%)
RPCA	–	0.528* (moderate)	–	0.13* (20.3%)	–	0.18 (25.5%)
LPCA	–	0.772** (good)	–	−0.020 (−2.8%)	–	0.15 (18.3%)
SSS	0.937*** (excellent)	0.697** (moderate)	−0.02 (−3.0%)	0.03 (20.1%)	0.04 (16.5%)	0.09 (53.1%)
StS	0.765** (good)	0.673** (moderate)	−0.02 (−2.3%)	−0.004 (2.7%)	0.06 (23.7%)	0.10 (30.3%)
RTS	–	0.902*** (excellent)	–	0.00 (5.3%)	–	0.06 (25.6%)
LTS	–	0.226 (poor)	–	0.05 (23.3%)	–	0.16 (52.8%)

Repeatability of PI measurements across intracranial vessels using 2D PC-MRI and 4D flow, assessed using ICC, paired *t*-tests, and Bland-Altman statistics. Significance indicators by the ICCs indicate *p-*value of ICC analysis, indicators by mean difference values indicate *p*-value of paired *t*-test: *p < 0.05, **p < 0.01, ***p < 0.001. Mean differences and standard deviations are reported in absolute values as well as the corresponding proportions (relative to scan 2 values). RICA/LICA, right/left internal carotid artery; BA, basilar artery; RMCA/LMCA, right/left middle cerebral artery; RACA/LACA, right/left anterior cerebral artery; RPCA/LPCA, right/left posterior cerebral artery; SSS, superior sagittal sinus; StS, straight sinus; RTS/LTS, right/left transverse sinus; s1/s2, scan 1/scan 2.

**Table 3:** Test-retest repeatability of mean flow.

Vessel	Intra-class correlation coefficient		Mean difference (s2-s1, ml/s)		Standard deviation of difference (ml/s)	
	2D	4D	2D	4D	2D	4D
RICA	–	0.547* (moderate)	–	0.37 (20.7%)	–	1.18 (54.1%)
LICA	–	0.700* (moderate)	–	0.00 (6.1%)	–	0.85 (37.8%)
BA	–	0.829** (good)	–	0.38 (19.2%)	–	0.40 (23.3%)
RMCA	0.674* (moderate)	0.411 (poor)	−0.17 (−4.4%)	−0.41 (−8.1%)	0.72 (25.7%)	0.68 (22.4%)
LMCA	–	0.571* (moderate)	–	−0.03 (9.6%)	–	0.84 (40.7%)
RACA	0.779** (good)	0.279 (poor)	−0.04 (−1.6%)	−0.12 (11.6%)	0.26 (23.0%)	0.54 (80.4%)
LACA	0.645* (moderate)	−0.160 (poor)	0.08 (15.3%)	−0.09 (6.0%)	0.28 (43.5%)	0.58 (42.3%)
RPCA	–	0.046 (poor)	–	0.19 (43.1%)	–	0.57 (98.2%)
LPCA	–	0.353 (poor)	–	−0.27* (−11.6%)	–	0.35 (21.5%)
SSS	0.964*** (excellent)	0.860*** (good)	0.15 (3.0%)	0.30 (7.0%)	0.22 (4.3%)	0.65 (13.8%)
StS	0.711** (moderate)	0.642* (moderate)	0.05 (2.3%)	−0.07 (−4.6%)	0.27 (20.1%)	0.39 (25.5%)
RTS	–	0.946*** (excellent)	–	−0.32 (−6.3%)	–	−0.73 (13.2%)
LTS	–	0.854*** (good)	–	−0.29 (1.9%)	–	1.19 (26.2%)

Repeatability of mean flow (ml/s) measurements across intracranial vessels using 2D PC-MRI and 4D flow, assessed using ICC, paired *t*-tests, and Bland-Altman statistics. *p < 0.05, **p < 0.01, ***p < 0.001. Mean differences and standard deviations are reported in absolute values as well as the corresponding proportions (relative to scan 2 values). RICA/LICA, right/left internal carotid artery; BA, basilar artery; RMCA/LMCA, right/left middle cerebral artery; RACA/LACA, right/left anterior cerebral artery; RPCA/LPCA, right/left posterior cerebral artery; SSS, superior sagittal sinus; StS, straight sinus; RTS/LTS, right/left transverse sinus; s1/s2, scan 1/scan 2.

**Figure 4:**
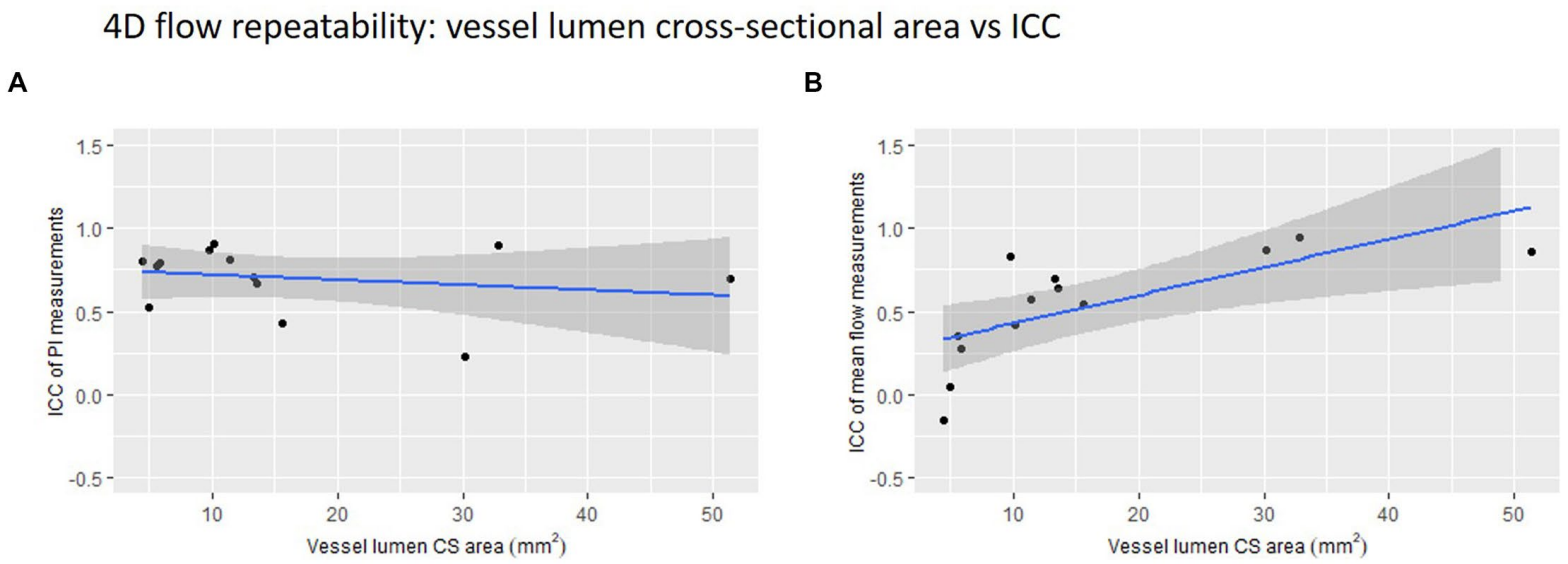
4D flow repeatability of **(A)** pulsatility index (PI) and **(B)** mean flow measurements plotted against vessel lumen cross-sectional (CS) area. The intraclass correlation coefficient is used here as a measure of repeatability.

We found a small, non-significant negative correlation between vessel lumen area and 4D flow-derived PI measurement ICC (*r* = −0.21, *p* = 0.485) (Figure 4). Conversely, we found a significant positive correlation between lumen area and 4D flow-derived mean flow measurement ICC (*r* = 0.70, *p* = 0.008).

The mean difference between inflow and cerebral flow was −0.57 ± 1.03 mL/s at visit 1 and 0.14 ± 1.13 mL/s at visit 2. A negative difference indicates higher measured cerebral flow than inflow and a positive difference indicates the reverse. These values represent mass conservation errors between 1 and 5% of average inflow, which we feel are acceptably low.

### Intra-rater reliability

For repeated analysis of the relevant 2D data, RMCA PI and mean flow measurements produced ICC scores of 0.991 (excellent) and 0.975 (excellent), and mean (± SD) differences of 0.01 ± 0.03 (*p* = 0.136) and 0.01 ± 0.12 mL/s (*p* = 0.823), respectively. SSS PI and mean flow measurements produced ICC scores of 0.999 (excellent) and 0.986 (excellent), and mean differences of 0.00 ± 0.05 (*p* = 0.299) and 0.12 ± 0.01 mL/s (*p* = 0.002), respectively.

For repeated analysis of the 4D flow data, RMCA PI and mean flow measurements produced ICC scores of 0.877 (good) and 0.459 (poor), and mean differences of −0.02 ± 0.08 (*p* = 0.405) and 0.39 ± 0.70 mL/s (*p* = 0.094), respectively. SSS PI and mean flow measurements produced ICC scores of 0.906 (excellent) and 0.723 (moderate), mean differences of −0.01 ± 0.06 (*p* = 0.659) and 0.57 ± 0.84 mL/s (*p* = 0.049), respectively.

### Method comparison

**Figure 5:**
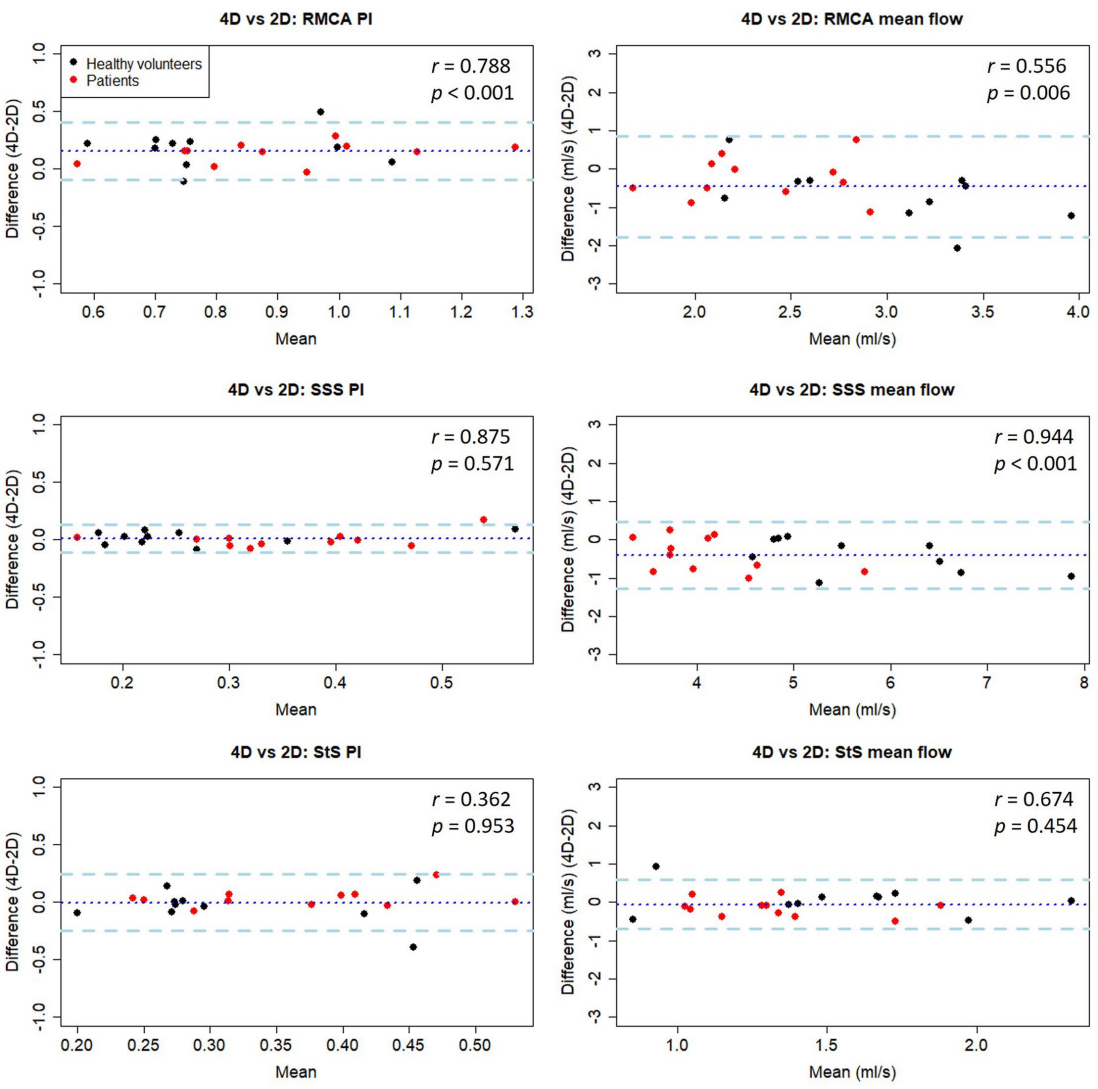
Bland-Altman plots of PI and mean flow measurements compared between 4D and 2D flow measurement methods in healthy volunteers. Central dotted line indicates mean difference between visits, outer dashed lines indicate 95% limits of agreement. RMCA, right middle cerebral artery; RACA/LACA, right/left anterior cerebral artery; SSS, superior sagittal sinus; StS, straight sinus.

On average, PI measured using 4D flow was of lower magnitude than that obtained by the 2D method for four of the five vessels measured (*p* < 0.05 for RMCA and RACA; Table 4; Figure 5). Moderate to strong PI correlation was seen in all vessels (*r* = 0.63–0.89, *p* < 0.05) except the StS (*r =* 0.15, *p* = 0.660). Conversely, the 4D method overestimated mean flow compared with the 2D method for four of the five vessels (*p* < 0.05 for RMCA, RACA, and SSS). Arterial flow measurements showed poor to moderate correlation between methods (*r* = 0.22–0.60, *p* > 0.05) while venous flow measurements showed moderate to excellent correlation (*r* = 0.69–0.93, *p* < 0.05).

**Table 4:** Comparison of 2D and 4D flow assessment of PI and mean flow.

Vessel	Subject group	Mean difference (4D – 2D)		Standard deviation of difference	
		PI	Mean flow (ml/s)	PI	Mean flow (ml/s)
RMCA	HV	−0.17**	0.72**	0.16	0.72
	P	−0.15***	0.17	0.09	0.50
RACA	HV	−0.14*	0.34*	0.20	0.42
LACA	HV	−0.11	0.35	0.18	0.54
SSS	HV	−0.02	0.46**	0.06	0.44
	P	0.00	0.34*	0.07	0.45
StS	HV	0.03	−0.04	0.15	0.38
	P	−0.03	0.16	0.09	0.24

2D/4D method comparisons of PI and mean flow measurements across intracranial vessels, assessed using paired *t*-tests and Bland-Altman statistics. *p < 0.05, **p < 0.01, ***p < 0.001. RMCA, right middle cerebral artery; RACA/LACA, right/left anterior cerebral artery; SSS, superior sagittal sinus; StS, straight sinus; HV, healthy volunteers; P, patients (with sporadic small vessel disease).

### Method comparison (patients)

As seen in the healthy subjects, PI measurements were higher using the 2D method (*p* < 0.05 for RMCA), with moderate to good correlation between methods (*r* = 0.64–0.88, *p* < 0.05). Mean flow measurements were higher using the 4D method (*p* < 0.05 for SSS), with poor to moderate correlation (*r* = 0.44–0.57, *p* > 0.05).

Combining data collected from both healthy and patient groups, we found good PI measurement correlation between methods for the RMCA (*r* = 0.79) and SSS (*r* = 0.88), but not the StS (*r* = 0.36) (Table 4 and Figure 5). As before, arterial PI measurements were higher using the 2D method (*p* < 0.001) while this was not the case for the SSS (*p* = 0.571) or StS (*p* = 0.953). Mean flow measurement correlation between methods was moderate for the RMCA (*r* = 0.56) and StS (*r* = 0.67), and excellent for the SSS (*r* = 0.94). The 4D method measured higher for the RMCA (*p* = 0.006) and SSS (*p* < 0.001). We saw no noticeable difference in inter-method variation between healthy and patient groups.

### Subject comparison

**Figure 6:**
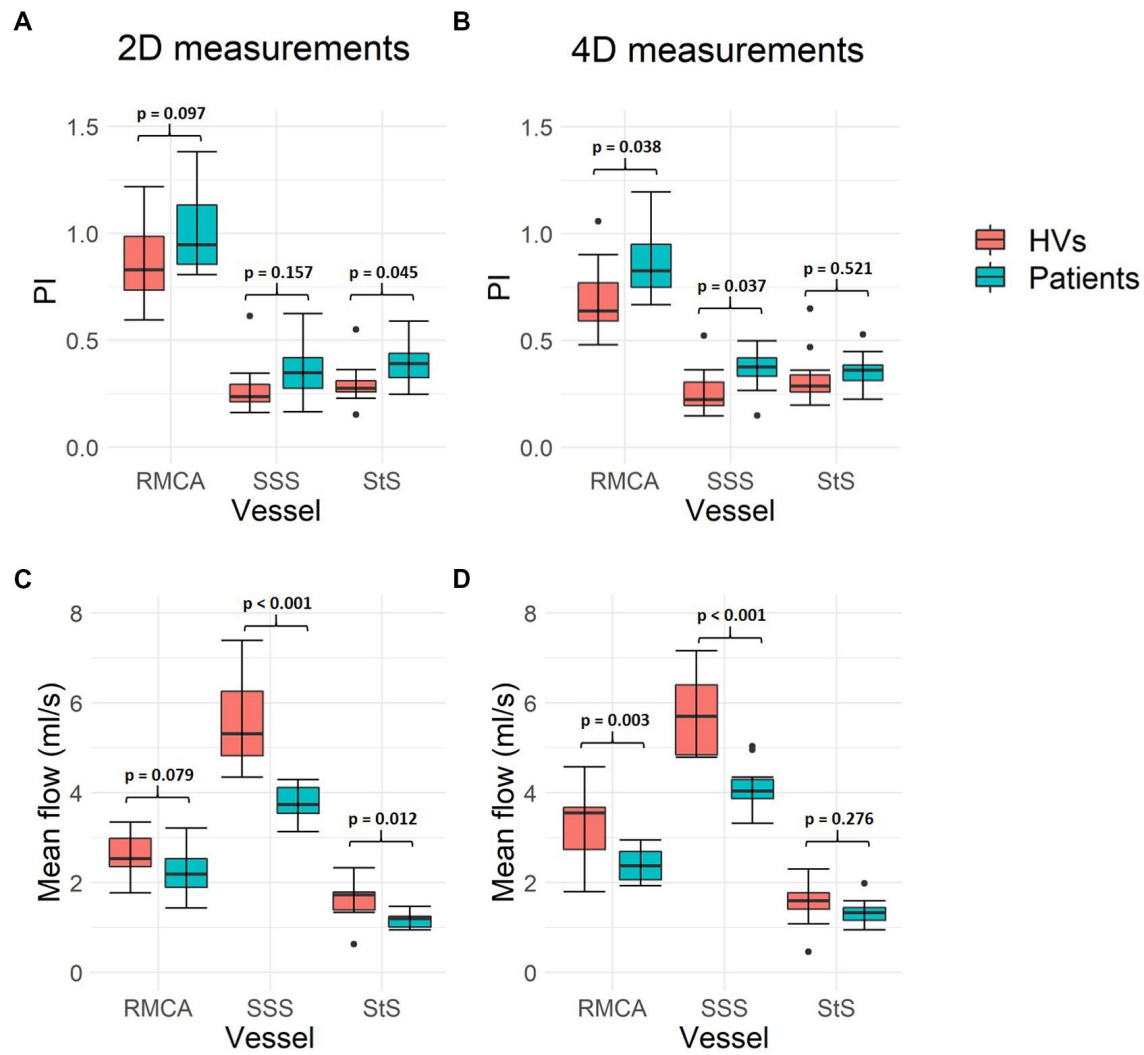
Intracranial flow measurement differences between healthy volunteers and patients with SVD. **(A,B)** Show mean flow measurement differences across the three vessels assessed, using both 2D PC-MRI and 4D flow, respectively. **(C,D)** Show pulsatility index (PI) measurements. Paired sample *t*-test *p*-values are shown for each pair of measurements. Black data points represent outliers. RMCA, right middle cerebral artery; SSS, superior sagittal sinus; StS, straight sinus; HVs, healthy volunteers.

PI measurements were higher in patients with SVD compared to healthy volunteers across all three vessels assessed (Figure 6), with both 2D (RMCA: *p* = 0.097; SSS: *p* = 0.157; StS: *p* = 0.045) and 4D (RMCA: *p* = 0.038; SSS: *p* = 0.037; StS: *p* = 0.521) methods. Meanwhile, mean flow measurements were lower in patients across all vessels, with both 2D (RMCA: *p* = 0.079; SSS: *p* < 0.001; StS: *p* = 0.012) and 4D (RMCA: *p* = 0.003; SSS: *p* < 0.001; StS: *p* = 0.276) methods.

## Discussion

We scanned 11 healthy volunteers and 10 patients with stroke presentations of sporadic SVD with both the widely-used 2D PC-MRI (Shi et al., 2018a; Blair et al., 2020, 2021; Clancy et al., 2021) and the more advanced 4D flow MRI methods to assess (a) test-retest repeatability, (b) intra-rater reliability, (c) inter-method consistency of flow and pulsatility parameters in the cerebral arteries and veins, and (d) feasibility of 4D flow measurements in a clinically-relevant patient subgroup. We found that both 2D and 4D flow methods showed overall good repeatability across sessions and reliability between repeated ratings when measuring PI, and that our 4D processing method produced similar results to those gained from the higher-resolution 2D data, particularly for PI measurements. We also showed that measurements from the intracranial veins, vessels not often examined in this context, rated well across these different metrics.

### Repeatability

The 2D PC-MRI PI measurements showed mostly good test-retest repeatability, while the mean flow measurements showed mostly moderate repeatability—with smaller vessels (ACAs) showing lower agreement in both cases. As previously reported, 2D PC-MRI flow and pulsatility measurements (at 3 T) have excellent repeatability in vessels at the C2-C3 area (Sakhare et al., 2019) and slightly less (while still acceptable) in the main intracranial arteries (De Verdier and Wikström, 2016), the latter aligning with the findings presented here.

The 4D flow measurements showed acceptable PI repeatability and moderate mean flow repeatability. As the 4D scan allowed for the measurement of more cerebral vessels, we were able to assess considerably more data. These results show the potential of 4D flow to evaluate and compare differences in blood flow across a broad range of vessels in a single scan. While the paired *t*-tests comparing the PI and mean flow results between scanning sessions visits sometimes produced statistically significant *p*-values, indicating the mean difference between visits was not zero, the small sample size means we should not rely too heavily on these results. It is promising to see that the intracranial veins demonstrated acceptable repeatability in flow rate and (in most cases) PI measurement. In the case of the transverse sinuses, the right vessel was nearly always dominant and easier to measure, while the left, however, was often small and exhibited fairly flat, low flow rates—leading to poor PI repeatability. This highlights the fact that some vessels are much easier to assess within the 4D volume than others and will vary between subjects. While we found little statistical evidence to suggest that poor PI measurement repeatability is related to vessel lumen size, we did confirm that flow measurements show higher repeatability in larger vessels.

Overall, it seems the 4D method is more reliable for PI measurements than it is for flow measurements. We speculate that this is due to the former being mostly dependent on the shape of the velocity waveforms captured within the segmented pixels (that are often largely similar across pixels), while the latter is also dependent on the number of these segmented pixels and the specificity of their selection. Measurements from several (three, for example) adjacent slices, with the mean flow value calculated across these taken, is likely a sensible approach to reduce this variability (Schrauben et al., 2015).

In contrast to the high repeatability of ICA flow and PI measurements using 2D PC-MRI reported previously, our 4D results showed lower repeatability in the ICAs. This is likely due to the limited coverage of the 4D volume, preventing us from capturing the SSS, circle of Willis and ascending straight sections of the ICAs in one acquisition. Limited to just the tortuous carotid siphon, flow plane placement on the ICAs presented a challenge and likely led to the bilateral ICC differences between these vessels.

### Reliability

With repeated analysis by the same observer, the 2D data processing was very reliable in both arterial and venous vessels. This may reflect that the same image slices were processed each time, perhaps reducing scope for variation. The 4D data proved to be less reliable, as the volume had to be resliced before the masks could be drawn, allowing for more variation in flow measurements. In future experiments, and with more experience, a more standardised and established 4D plane selection protocol, as well as semi-automatic segmentation methods such as intensity-based thresholding (Dunas et al., 2019), may help reduce this variation. These will be important for standardising future clinical research to avoid apparent variation in vessel sampling between studies or centres being attributed to true between-sample differences. As with the test-retest repeatability, the PI measurements showed higher intra-rater reliability than the mean flow measurements.

The multicentre reproducibility, test-retest reliability, and inter-rater dependence of blood flow from 4D flow scans in healthy volunteers have previously been shown to be strong (Wen et al., 2019). The results presented here somewhat conform to these previous findings, although not fully as the authors found higher reproducibility of blood flow measurements than found here—possibly due to the use of advanced k-t MR acceleration and specialised flow analysis software (which allowed for semi-automatic placement of ROIs and likely reduced variability).

### Inter-method conformity

The 2D results often showed higher arterial PI measurements than their 4D counterparts. This is likely due to the higher spatial and temporal resolutions of the 2D method, potentially allowing pulsatile flow (including the peak) to be more accurately sampled. The longer acquisition time of the 4D flow compared to a single 2D scan may have led to temporal smoothing if flow waveforms were inconsistent between heartbeats, potentially reducing PI measurement due to the reconstructed data representing an average across heartbeats (Wahlin et al., 2013). Mean flow, however, was often measured higher using the 4D method, likely because the lower spatial resolution can lead to partial volume effects and overestimation of flow (Peng et al., 2015). Unfortunately, increasing 4D flow spatial resolution would increase scan time and decrease SNR. Partial volume correction methods have been suggested previously for 2D data (Tang et al., 1995) and more recently for 4D data (Bouillot et al., 2018), which will be considered in future work.

Although the difference in 2D and 4D measurements was statistically significant in some vessels, the small sample size should again be taken into consideration (as a single large difference in measurements could have strong leverage on the mean) when interpreting these results. Overall, the results shown here are promising in that 4D flow appears to perform relatively close to the standard set by its 2D predecessor when it comes to PI measurement, although more apparent differences exist with mean flow measurements.

Several studies have compared 4D flow with 2D PC-MRI and found strong correlations between the techniques when measuring flow in the internal carotid (Schrauben et al., 2015), basilar (Meckel et al., 2013), and cerebral arteries (Meckel et al., 2013; Wahlin et al., 2013; Dunas et al., 2019). However, this is the first time that this many intracranial arteries and veins have been examined using single 4D flow acquisitions across repeat visits, with comparisons between 2D and 4D methods. Importantly, we showed that there exists good agreement between methods when quantifying flow and pulsatility in intracranial veins rarely examined using these techniques. We also showed relatively small differences and high correlations in PI measurement when assessing older SVD patients. This is a promising finding for the future of SVD research.

The variation in flow measurement differences seen between methods was larger than that of a previous, similar study (Wahlin et al., 2013) in which SDs ranged from 0.21 to 0.43 mL/s across a range of cerebral arteries (compared to SDs ranging from 0.24 to 0.72 mL/s here). This may be due, in part, to their use of a novel, in-house-developed software to segment the vessels, utilising composite complex difference images and region-growing—as opposed to the manual segmentation carried out here that may have introduced more variation due to potential lack of consistency.

### Healthy vs. SVD group

We also showed that patients with SVD exhibit lower blood flow and increased pulsatility in both arteries and veins compared to healthy volunteers. Interestingly, these differences were more often statistically significant in the 4D data. These results align with previous literature on SVD, as vessel stiffening and reduced flow are commonly seen in patients with the disease (Shi et al., 2016, 2018b; Wardlaw et al., 2019). However, it is important to note that these subject groups were not age-matched [with age having previously been shown to be positively associated with pulsatility (Lefferts et al., 1985)] and there are other confounding factors, such as blood pressure (Aribisala et al., 2014), at play. Regardless, it is encouraging to see these findings in both the 2D and 4D data.

### Comments on 4D flow practicality

For the purposes of examining small cerebral vessels, 1 mm isotropic voxels are suboptimal [as even the 2D method’s in-plane resolution of 0.8 × 0.8 mm^2^ has been suggested to be too low for vessels <3 mm in diameter (Fukuyama et al., 2017; Isoda and Fukuyama, 2022)], but the protocol optimisation phase of our study indicated that increasing the isotropic resolution decreased SNR considerably whilst providing little in the way of increased flow measurement conformity to 2D results. 2D PC-MR images, on the other hand, can be acquired with high in-plane resolution and thick slices to maintain high SNR—provided the vessels are reasonably straight.

While the acquisition times of the 4D flow scans were substantially longer than the 2D scans (11–17 min vs. 1–3 min) the greater coverage in a single acquisition minimises the scan setup time and does not require *a priori* identification of specific vessels, which can be extremely challenging due to anatomical variability, potentially leading to repeated scans.

### Limitations

A limitation of the study is the small sample sizes used (11 healthy volunteers and 10 SVD patients). This meant that the results were more sensitive to outliers, allowing for a single pair of mismatched measurements to notably reduce the repeatability of a vessel. Secondly, the relatively long 4D flow scan times meant that only single-*v*_enc_ acquisition could be obtained whilst keeping scan times feasible. To capture all possible blood flow velocities, this was set to 100 cm/s and thus will have reduced the sensitivity to slower velocities in the veins and arterial lumen perimeters. It is likely that some of the lower ICC values are attributable to velocity measurement errors caused by this high *v*_enc_, especially so in smaller vessels. In future studies, *v*_enc_ could be set to the vessel type of interest (i.e. arteries, veins, cerebrospinal fluid) for higher SNR. Alternatively, a lower *v*_enc_ than used here could be used for all vessel types, with increased reliance on potential phase-unwrapping, or perhaps a multi-*v*_enc_ approach (Schnell et al., 2017; Nakaza et al., 2022). Meanwhile, the *v*_enc_ chosen for the 2D RMCA scans led to wraparound in some cases (due to being too low) and therefore required correction.

We found that smaller vessels such as the PCAs, and tortuous vessels such as the ICAs, provided the greatest challenge for 4D flow assessment, due to limited spatial resolution and challenging flow plane placement caused in part by limited volume coverage of vessels, respectively. A volume that captured the proximal ICAs (pre-siphon) would likely have led to less challenging ICA analysis and thus more repeatable results. Furthermore, although it would have been beneficial to assess repeatability in the patient group, time limitations and the nature of the SVD study prevented this.

### Conclusion

In conclusion, the 4D flow MRI protocol resulted in a clinically feasible scan time (approximately 10–15 min), with an isotropic spatial resolution of 1.0 mm, capable of assessing the major vessels of the brain (and even some smaller branches) with good PI repeatability, intra-rater reliability and accuracy in relation to 2D PC-MRI. In fact, for arterial PI measurements, 4D flow often demonstrated better test-retest repeatability than 2D PC-MRI, suggesting we can be confident in using our 4D flow protocol to measure pulsatility across the major cerebral vessels. Mean flow rate measurements of these intracranial vessels exhibit more test-retest variability in both methods, however. We have shown that the sequence is feasible in the evaluation of intracranial veins thus far rarely examined, which is an important finding. Recent and future methodological advances will likely extend 4D flow’s application to the smaller vessels implicated in neurodegenerative pathologies such as cerebral small vessel disease. Our findings confirm and quantify its reliability, and should therefore aid the design of future clinical studies and trials.

## Data availability statement

The original contributions presented in the study are included in the article/Supplementary material, further inquiries can be directed to the corresponding author.

## Ethics statement

The studies involving human participants were reviewed and approved by South East Scotland Research Ethics Committee. The patients/participants provided their written informed consent to participate in this study.

## Author contributions

AM, MT, JW, and IM were responsible for study concept and design. NJ was responsible for sequence design. AM, MT, and IM were responsible for data collection. AM and MS were responsible for data analysis. AM, MT, MS, NJ, JW, and IM were responsible for drafting the manuscript and figures. All authors contributed to the article and approved the submitted version.

## Funding

The authors disclosed receipt of the following financial support for the research, authorship and/or publication of this article: AM and MRI scanning was funded by Medical Research Scotland, Siemens Healthcare, and the Medical Research Council through the UK Dementia Research Institute. Siemens Healthcare provided the work-in-progress 4D flow MRI pulse sequence and NJ reviewed the draft manuscript for technical accuracy. The funders were not otherwise involved in the study design, collection, analysis, interpretation of data, the writing of this article, or the decision to submit it for publication. MT is funded by NHS Lothian Research and Development Office. MS is funded by the European Union Horizon 2020 project No. 666881, “SVDs@Target” and Fondation Leducq (ref no. 16 CVD 05). JW is funded by the UK Dementia Research Institute which receives its funding from DRI Ltd., funded by the UK Medical Research Council, Alzheimer’s Society and Alzheimer’s Research UK. The MR scanner was funded by the Wellcome Trust 104916/Z/14/Z, Dunhill Trust R380R/1114, Edinburgh and Lothians Health Foundation 2012/17, Muir Maxwell Research Fund, Edinburgh Imaging, and the University of Edinburgh. The authors acknowledge support from the Scottish Funding Council and Chief Scientist Office through the Scottish Imaging Network: A Platform for Scientific Excellence (SINAPSE). The 4D flow sequence was provided by Siemens (NJ) as part of a technical research collaboration. The work is that of the authors and does not reflect the views of the funders. For the purpose of open access, the author has applied a CC BY public copyright license to any Author Accepted Manuscript version arising from this submission.

## Conflict of interest

NJ was employed by Siemens Medical Solutions USA, Inc. The authors declare that this study received funding from Siemens Healthcare, which had the following involvement in the study: provision of work-in-progress 4D flow MRI pulse sequence; NJ reviewed the draft manuscript for technical accuracy. While AM’s PhD was partially funded by Siemens, all research was carried out without commercial influence.

The remaining authors declare that the research was conducted in the absence of any commercial or financial relationships that could be construed as a potential conflict of interest.

## Publisher’s note

All claims expressed in this article are solely those of the authors and do not necessarily represent those of their affiliated organizations, or those of the publisher, the editors and the reviewers. Any product that may be evaluated in this article, or claim that may be made by its manufacturer, is not guaranteed or endorsed by the publisher.

## Footnotes

^1^ https://imagej.nih.gov/ij/

^2^ https://www.mathworks.com/matlabcentral/fileexchange/32032-extract -slice-from-volume

## Supplementary material

SUPPLEMENTARY DATA SHEET 1 2D phase-contrast MRI flow and pulsatility measurements for patients with SVD.

SUPPLEMENTARY DATA SHEET 2 2D phase-contrast MRI flow and pulsatility measurements for healthy volunteers (visit 1).

SUPPLEMENTARY DATA SHEET 3 2D phase-contrast MRI flow and pulsatility measurements for healthy volunteers (visit 1 - repeated analysis).

SUPPLEMENTARY DATA SHEET 4 2D phase-contrast MRI flow and pulsatility measurements for healthy volunteers (visit 2).

SUPPLEMENTARY DATA SHEET 5 4D flow MRI flow and pulsatility measurements for patients with SVD.

SUPPLEMENTARY DATA SHEET 6 4D flow MRI flow and pulsatility measurements for healthy volunteers (visit 1).

SUPPLEMENTARY DATA SHEET 7 4D flow MRI flow and pulsatility measurements for healthy volunteers (visit 1 - repeated analysis).

SUPPLEMENTARY DATA SHEET 8 4D flow MRI flow and pulsatility measurements for healthy volunteers (visit 2).
